# The Third Konstantin Ivanov Intercontinental Magnetic Resonance Conference on Methods and Applications ICONS-3

**DOI:** 10.1007/s00723-021-01441-z

**Published:** 2021-10-24

**Authors:** G. Buntkowsky, D. Abergel, P. K. Madhu

**Affiliations:** 1 Institute of Inorganic and Physical Chemistry, Technical University of Darmstadt, 64287 Darmstadt, Germany; 2 Laboratoire des Biomolécules, Département de chimie, École Normale Supérieure, PSL University, CNRS, Sorbonne Université, 75005 Paris, France; 3 TIFR Centre for Interdisciplinary Sciences, Tata Institute of Fundamental Research Hyderabad, 36/P Gopanpally Village, Ranga Reddy District, Hyderabad, 500107 India

**Figure:**
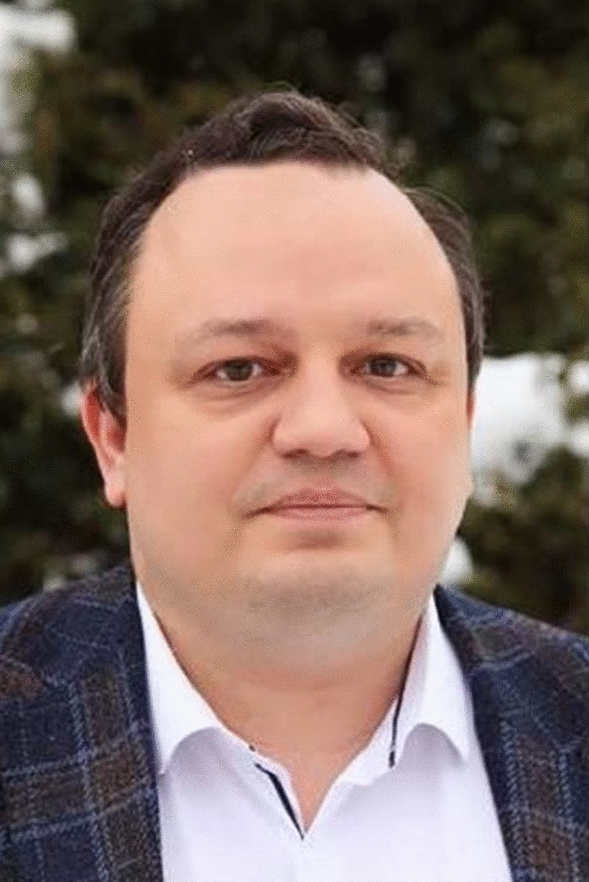
In memory of, Prof. Dr. Konstantin L’vovich Ivanov. 10 January 1977 – 05 March 2021

ICONS-3, organized during September 01–03, 2021, was the third edition of the on-line magnetic resonance conference series called Konstantin Ivanov Intercontinental Magnetic Resonance **S**eminary, named after our untimely deceased colleague and friend. The ICONS conferences are an off-shoot of the weekly Intercontinental NMR Seminar Series that started on April 8, 2020. This seminar series enables communication and dissemination of research ideas among the magnetic research community especially in the times of the COVID-19 pandemic. In the frame-work of the ICONS series, until now, about one-hundred scientists from five different continents have presented their recent results. While the weekly seminar series gives both early-stage and experienced researchers an opportunity to give seminar talks and interact with colleagues from all over the world, the ICONS-3 conference is a platform for experienced researchers. The ICONS-3 conference attracted registrations from more than 220 researchers from over 30 countries covering 4 continents and spanning 14 time zones.

The ICONS seminar series is open to all areas of magnetic resonance and covers the full range of Magnetic Resonance, i.e., EPR, NMR, MRI, and their various hybrids. The first ICONS conference in 2020 (see report in APMR [1] for details) covered the full band-width of magnetic resonance, and the second ICONS in spring 2021 focused on fields where the interaction of electron and nuclear spins plays a pivotal role (see report in APMR [2] for details).

Owing to the untimely death of our friend and co-organizer, Prof. Dr. Konstantin Ivanov, (known to many as Kostya), we decided to devote a part of the ICONS3 to his memory. Kostya, who had a very broad range of interests in the field of magnetic resonance, sadly passed away on March 5, 2021 as a victim of COVID-19. He was instrumental in initiating the ICONS seminar and conference series. To honor his contributions to science, we decided to invite about one third of the speakers among well-known scientists who were former mentors, colleagues, and collaborators of Kostya and asked them to give in their presentations not only information about their latest research but also display some facets of Kostya’s many contributions to their fields of magnetic resonance.

The presentations covered a wide range of topics, including recent advances in the fields of NMR of proteins and nucleic acids, quadrupolar nuclei and ultra-wide-line NMR to time-resolved NMR of biomolecular systems, the development and application of hyperpolarization techniques like Chemically Induced Nuclear Polarization, Dynamic Nuclear Polarization and Parahydrogen-Induced Polarization, to the potential of hyper-polarized triplet states as spin labels for EPR to the investigation of radical pair reactions.

**Alexandra Yurkovskaya**, ITC, Novosibirsk, reported results on Time Resolved Chemically Induced Dynamic Nuclear Polarization (TR-CIDNP), and how it can be exploited to learn details of the chemical reaction mechanism and the dynamics of fast radical reactions. In her presentation, she gave special emphasis on the linear relationship between the signal intensities observed in TR-CIDNP spectra and the value of the hyperfine interaction constants in short-lived states. This relationship, which was explained by Konstantin Ivanov in 2011, is now widely used to characterize the spin-density distribution in transient radicals in multistage reactions at biologically relevant conditions.

**Geoffrey Bodenhausen**, Paris, reported on recent findings in the field of dissolution DNP experiments. In these experiments, a microwave-dependent change of the proton relaxation times was observed in the build-up curves of the nuclear spin polarization, resulting in an extension of the nuclear coherence lifetime upon switching off of the microwave. After the presentation of the effect, he discussed the potential to employ this effect to measure the longitudinal relaxation times of the electron spins.

**Matvey Fedin**, ITC, Novosibirsk, discussed recent results on the measurement of spin–spin distances by pulsed dipolar (PD) EPR spectroscopy employing photoexcited triplet states as spin labels. Owing to their very high non-thermal polarization, which results from their optical excitation, they exhibit far superior sensitivity than common stable–radical-type spin labels.

**Hans-Martin Vieth**, FU Berlin and ITC, Novosibirsk, first gave an overview about the historic development and current state of the art of the role of Level-Anti-Crossings (LACs) in manipulating the population of spin levels. In this overview, he focused on experimental schemes for utilizing LACs in optics and magnetic resonance. Then he discussed a number of important contributions of Konstantin Ivanov to the field of LACs, who set the frame-work for understanding the spin dynamics of hyperpolarization experiments like SABRE as a result of level crossings of spin levels.

**Robert Kaptein**, Utrecht, reported on NMR investigation of DNA binding to proteins and in particular on DNA recognition of gene regulatory proteins and sliding of DNA fragments along the protein. After revealing the structure of the DNA–protein complex, he demonstrated how the sliding rate of the fragment on the protein can be determined from the analysis of the NMR line-width and compared the result to data from laser spectroscopy.

**Dmitry Budker**, Mainz, first discussed recent developments in zero- to ultralow-field (ZULF) NMR and future prospects in single-molecule spectroscopy with a single-spin sensor. Then he explained the potential of nuclear spins to search for galactic dark matter and explained some details of how NMR is utilized in the Cosmic Axion Spin Precession Experiments (CASPEr) program as a potential monitor of dark matter.

**Olivier Lafon,** Lille, introduced novel methods to probe the local environment of quadrupolar nuclei, such as ^17^O, ^47,49^Ti, ^67^Zn, and ^95^Mo and others in solids. These methods combine robust pulse sequences to transfer the polarization of protons to quadrupolar nuclei over a wide range of magic-angle spinning (MAS) frequencies with dynamic nuclear polarization (DNP) to detect the nuclei on or near the surface of heterogeneous catalysts, inorganic nanoparticles, or organic solids with very high sensitivity.

**Marco Tessari**, Nijmegen, showed an exciting application of reversible para-hydrogen-induced polarization (SABRE) to boost the sensitivity of NMR in analytical chemistry. The reversible association of the analytes to the iridium catalysts selectively hyperpolarizes the analytes and permits their NMR detection down to nanomolar concentrations, while removing the signal background originating from the other species in solution.

**Robert Schurko**, NHMFL Tallahassee, discussed the challenges of ultra-wide-line solid-state NMR spectroscopy of unreceptive isotopes and how these challenges are resolved by new experimental schemes, including improved pulses, pulse sequences, methodologies, and specialized hardware developments. In particular he reported details on various applications of the broadband adiabatic inversion-cross polarization (BRAIN-CP) methods in NMR spectroscopy and relaxometry, novel numerical evaluation tools for data analysis of these systems and indirect detection schemes.

**Robert Tycko**, NIH, Bethesda, reported on new developments and recent applications of low-temperature magic-angle spinning and dynamic nuclear polarization to obtain two-dimensional solid-state NMR measurements of frozen solutions of peptides, proteins, and other biological molecules at sub-millimolar concentration. The implementation of rapid negative temperature jumps, and rapid freezing techniques enabled him to investigate transient intermediate states of biomolecular systems that exist under native conditions only on the order of 10 ms by solid-state NMR. In addition, the advantages of new tri-radicals for cross-effect DNP were discussed.

**Robert Konrat**, Vienna, discussed new developments in the structural characterization of intrinsically disordered proteins (IDPs) by a combination of NMR spectroscopy and novel computational protein sequence analysis tools.

**Kiminori Maeda**, Saitama, gave an exciting overview about recent advances in the investigation of radical pair reactions in photo-chemistry by the Magnetic Field Effect (MFE) and the Reaction Yield Detected Magnetic Resonance (RYDMR). Employing a time-resolved version of the MFE called Switched External Magnetic Field (SEMF), he could measure back-ground free the decay kinetics of the radical pairs.

**Nikita Lukzen**, ITC, Novosibirsk, introduced into the application of multi-frequency nuclear magnetic resonance as an efficient tool to investigate heterospin complexes of Europium compounds in solution to determine the paramagnetic shifts of the ligands and the equilibrium constants of the systems and discussed the potential of these Europium complexes as chemical-shift thermometer.

**Lewis E Kay**, Toronto, finally, discussed a number of NMR tools developed by his group for the characterization of intrinsically disordered protein regions (IDRs). Employing these tools, they could identify ATP and side-chain interactions with an RNA-binding protein (CAPRIN1) that influence the phase behavior of the protein.

**Organization and Future Developments**

The conference was organized by Daniel Abergel (ENS Paris, France), Gerd Buntkowsky (TU, Darmstadt, Germany), and P. K. Madhu (TIFR Hyderabad, India). Suman Saurav and Sreenidhi, TIFR Hyderabad, provided technical assistance. The conference and seminar series were sponsored by Alexander von Humboldt Foundation, Wiley, Springer, HyperSpin, and Adani. Following the scheme of a general MR conference in summer alternating with a specialized conference on cutting-edge topics in winter, there are already plans for a specialized ICONS4 in winter of 2022. For updates and the schedule of upcoming talks see the home page of the meeting ICONS-Seminary.
